# Autoantibodies Recognizing the Amino Terminal 1-17 Segment of CENP-A Display Unique Specificities in Systemic Sclerosis

**DOI:** 10.1371/journal.pone.0061453

**Published:** 2013-04-22

**Authors:** Elvira Favoino, Liboria Digiglio, Giovanna Cuomo, Isabella E. Favia, Vito Racanelli, Gabriele Valentini, Federico Perosa

**Affiliations:** 1 Department of Internal Medicine (DIMO), University of Bari Medical School, Bari, Italy; 2 Department of Clinical and Experimental Internal Medicine, Second University of Naples, Naples, Italy; Cordelier Research Center, INSERMU872-Team16, France

## Abstract

Centromere-associated protein A (CENP-A), a common autoimmune target in a subset of systemic sclerosis patients, appears to have no role to explain why its corresponding auto-antibodies are more frequently found in the limited than the diffuse form of systemic sclerosis. Therefore, we investigated the fine specificity of anti-CENP-A antibodies as a first step to understanding their role in systemic sclerosis pathology. We focused on the amino-terminal portion of CENP-A spanning amino acids 1 to 17 (Ap^1-17^), which represents, along with Ap^17-30^, an immunodominant epitope of the protein. Peptide Ap^1-17^ was used to purify antibodies from 8 patients with systemic sclerosis. Anti-Ap^1-17^ antibodies specifically reacted with human CENP-A but did not cross-react with CENP-B or Ap^17-30^. Panning of a phage display peptide library with anti-Ap^1-17^ antibodies from 2 patients identified two novel, partially overlapping motifs, <^5^Rx(st)xKP^10^> and <^9^KPxxPxR^15^> as the result of the alignment of specific phage clone insert sequences. Anti-Ap^1-17^ IgG from the 8 patients had different reactivities to isolated phage clone insert sequences. Scanning the Swiss-Prot database revealed a large number of different types of proteins containing the two Ap^1-17^ antigenic motifs. These data show that anti-CENP-A^1-17^ antibodies are generated independently from anti-CENP-B antibodies and display great heterogeneity in their specificity by recognizing different motifs within that peptide sequence. This finding, along with the widespread interspecies and human tissue distribution of the two motifs, suggests that the number of motif-expressing proteins which can be the potential target of these antibodies is markedly higher than that estimated from the peptide-based epitope spreading model.

## Introduction

Systemic sclerosis (SSc) is a disabling and incurable connective tissue disease with an unknown pathogenesis [1], [2]. In SSc, the combination of vascular abnormalities, collagen deposition and autoimmunity leads to widespread tissue and organ fibrosis.

Autoimmunity in SSc is demonstrated by the presence of an oligoclonal T cell response in the early stages of the disease [3] and of anti-nuclear antibodies (ANAs) in the sera of >95% of patients [2], [4]. ANAs recognize a wide variety of self-antigens, including DNA topoisomerase-I (topo-I or Scl70), RNA-polymerase III, Th/To and several heterologous centromeric-associated proteins (CENP-A, CENP-B and so on) [4]. Subsets of ANAs have been associated with different clinical manifestations and various degrees of SSc severity [4]–[6]. For instance, anti-topo-I antibodies (Abs) are associated with more diffuse cutaneous involvement [7], [8], pulmonary fibrosis [9], [10], renal involvement and, possibly, higher disease severity [2], whereas anti-CENP Abs are more common in patients with pulmonary hypertension [11], [12]. Anti-CENP Abs have also been found in over 80% of patients with limited cutaneous involvement, but in only 10% of patients with diffuse cutaneous involvement [2], [7], [9], [10]. Despite the association of these ANA subsets with different SSc clinical features, their direct or indirect role in the pathophysiology of SSc is for the most part unclear; two exceptions are anti-topo-I Abs, which recognize and activate fibroblasts [13], and anti-CENP-B Abs, which react with endothelial cells [14]. On the other hand, the pathogenetic role of anti-CENP-A Abs remains elusive.

One way of assessing the functional role of anti-CENP-A Abs is to define their fine specificity and determine whether proteins other than CENP are their real target or can prime them. Peptide scanning analysis of CENP-A with sera from anti-CENP Ab-positive patients identified the NH_2_-terminal 45 amino acid region as the reactive site [15], [16]. Within this region, two major antigenic determinants were found be the dominant epitopes of anti-CENP-A Abs, namely the region spanning residues 17 to 30 (CENP-A^17-30^) and that from amino acid 1 to 17 (CENP-A^1-17^) [17]. Within these two immunodominant epitopes, a mutational analysis study identified the motif GPXRX [18]. Since this motif was also expressed on the amino terminal portion of CENP-B (^2^GPXRX^6^), it was thought that this more abundant protein could also trigger anti-CENP-A Abs.

Using a different methodological strategy which makes use of a phage display peptide library (PDPL), we previously defined two amino acid contact sites (motifs) of anti-CENP-A^17-30^ Abs [19], different from GPXRX. One of these motifs (PTPxxGPxxR) was found to also be expressed by the forkhead BOX E3 transcription factor (FOXE3), which had never previously been described as a potential target of anti-CENP-A Abs.

In the present report we extend this analysis to the anti-CENP-A Abs that target the other immunodominant epitope, namely CENP-A^1-17^. Using anti-Ap^1-17^ IgG purified from the sera of SSc patients, we identified two new motifs and found that anti-CENP-A Ap^1-17^Abs display a great heterogeneity in their fine specificity that is almost unique for each patient, despite the fact that these Abs recognize the same segment of CENP-A.

## Materials and Methods

### Patients and controls

Frozen serum samples from 57 patients affected with SSc and satisfying the preliminary American College of Rheumatology and Canadian Scleroderma Research Group criteria for the classification of the disease [20], [21] were obtained from serum bank at the Department of Internal Medicine, University of Bari and at Department of Clinical and Experimental Internal Medicine, University of Naples. All subjects provided informed consent, and the study was approved by institutional review board at the respective institutions.

On the basis of the presence of anti-centromere Abs (ACA) and anti-topo-I (anti-Scl70) Abs in their sera, determined on a routine basis using the anti-CENP-B and –Scl70 ELISA kits (Orgentec Diagnostika GmbH, Germany), the patients were distinguished into three groups: (i) ACA^+^, patients pt1–pt30; (ii) Scl70^+^, pt31–pt50; and (iii) ACA^−^/Scl70^−^, pt51–pt57. No patient had both ACA and anti-Scl70 Abs. In addition, serum samples from 10 healthy blood donors (HBD) were obtained from the hospital blood bank.

### Reagents, antibodies and peptides

Electrophoresis reagents were purchased from Bio-Rad Laboratories (Segrate, Milan, Italy). Unless otherwise specified, other chemicals were purchased from BDH Merck (Poole Dorset, UK) or Sigma-Aldrich (St. Louis, MO, USA).

Polyclonal human IgG preparations for intravenous use (IVIG, Intratec®) were purchased from Biotest (Dreieich, Germany). Anti-Ap^17-30^ IgG were purified from serum of patient 1 (pt1) as previously described [19].

The anti-HLA class I monoclonal Ab (mAb) HC-10 and its specific peptide Qp-1a (QEGPEYWDRNT), corresponding to residues 54 to 64 of the heavy chain of HLA class I antigen [22] were used as controls throughout the experiments. Another control was the peptide CBp^1-13^ (M**GP**KRRQLTFREK), corresponding to the amino terminal portion of CENP-B (amino acids 1 to 13) and bearing the motif <GPXRX>, shared with CENP-A [18].

Horseradish-peroxidase (HRP) or fluorescein isothiocyanate (FITC)-conjugated xeno-Abs to human or mouse IgG (Fc portion) were purchased from Jackson Immunoresearch Laboratories (Avondale, PA, USA). An HRP-conjugated mouse mAb to bacteriophage M13 major coat protein product of gene VIII was purchased from GE Healthcare Life Sciences (Milan, Italy).

### Synthesis and conjugation of peptides

Peptides were synthesized by Primm (Milan, Italy). Their purity, determined by analytical reverse phase chromatography and mass spectrometry, ranged between 92.26% and 100%. Peptides were coupled to KLH by means of glutaraldheyde as previously described [23].

### Serological assays

The reactivity and specificity of Abs with peptide were assessed by indirect ELISA in binding and inhibition assays, as described [24], with minor modifications. Briefly, polyvinylchloride 96-well plates were incubated with 50 µl PBS containing 5 µg/ml KLH-conjugated peptide for 12 h at 4°C. Wells were washed once with PBS containing 0.05% Tween 20 (PBS-T20) and blocked with PBS containing 0.5% BSA (PBS-BSA).

In binding assays, serum samples (diluted 100 times in PBS-BSA) or known concentrations of affinity-purified anti-Ap^1-17^ Abs (50 µl) were added to the wells and incubated for 4 h at 25°C. Wells were washed three times with PBS-T20. Bound IgG were detected by sequential incubation with HRP-conjugated xeno-Abs to the Fc portion of human (or mouse) IgG (1 h incubation at 25°C) and *o*-phenylenediamine (0.5 mg/ml; 100 µl/well); color development was stopped by adding 100 µl 2 N H_2_SO_4_ and the absorbance at 490 nm was read with the Benchmark microplate reader (Bio-Rad Laboratories). Background binding was determined from the absorbance generated in wells with blocking solution alone. Specific binding was determined by subtracting the background absorbance from the absorbance in experimental wells. All sera were tested at the same time to permit comparisons.

For inhibition assays, wells were coated with KLH-conjugated peptide as described for the binding assays. Ab preparations were diluted in PBS-T20 to the lowest concentration that gave 80%–100% of maximal A 490 in binding assays, and mixed with an equal volume of PBS containing inhibitor (carrier-free peptide or recombinant CENP) in 2-fold serial dilutions, starting at 800 µg/ml. The Ab-peptide solution was incubated for 2 h at 25°C prior to being added to the wells (100 µl/well). After 4 h at 25°C, wells were washed and bound IgG was detected as described for the binding assay. Results were expressed as percentage of inhibition, calculated as ([A_490_ in absence of inhibitor-A_490_ in presence of inhibitor]/A_490_ in absence of inhibitor)×100.

### Affinity purification of human anti-CENP-A IgG

CENP-A-derived peptide 1-17 (Ap^1-17^; ^1^MGPRRRSRKPEAPRRRS^17^) was conjugated to AffiGel 15 (Bio-Rad Laboratories, Hercules, CA, USA) at a concentration of 2 mg/ml resin following the manufacturer's instructions and used to purify human anti-CENP-A IgG as previously described [19]. Briefly, 5 ml of ACA^+^ serum samples, selected on the basis of their having high binding avidity for Ap^1-17^ in ELISA, were diluted in an equal volume of PBS, repeatedly passed through a BSA-conjugated AffiGel 15 column, and then absorbed several times on an Ap^1-17^-conjugated column. Bound IgG were eluted, dialyzed overnight against PBS, and concentrated by lyophilization. Ab concentration was determined by UV absorption with 1.35 extinction coefficient at 280 nm for 1 mg/ml. The extent of contamination of samples by human serum albumin was assessed by SDS-PAGE, Coomassie brilliant blue staining and density scanning; the final concentration of IgG was corrected to reflect this contamination.

### Western blotting and immunofluorescence

Recombinant human CENP-A and CENP-B proteins (Diarect, Freiburg, Germany) were resolved by 12.5% SDS-PAGE under non-reducing conditions and transferred onto polyvinylidene fluoride (PVDF) Immobilin P filters (Millipore, Bedford, MA, USA), previously soaked in absolute methanol. After blockade of free protein-binding sites by a 2 h incubation in PBS-BSA, the filter was incubated with 2 µg/ml affinity-purified anti-Ap^1-17^ Abs. Serum from pt1 (or pt8) containing anti-CENP-A and CENP-B Abs (diluted 400 times in PBS-BSA) and IVIG were used as controls. After extensive washes, bound IgG was detected by the sequential addition of HRP-conjugated xeno-Abs to the Fc portion of human IgG and diaminobenzidine substrate solution. The experiment was performed three times independently.

Immunofluorescence assays on HeLa cells were performed with purified anti-Ap^1-17^ IgG exactly as described [19].

### Panning of a PDPL

An M13-filamentous PDPL expressing random 12-mer linear peptides was purchased from New England Bio Labs (Beverly, MA, USA). Its characteristics have been previously described [22]. The PDPL was panned to identify the antigenic determinants of the anti-Ap^1-17^ Abs from two SSc patients, chosen on the basis of the highest yield after affinity purification, as previously described [19]. The panning of the peptide library with affinity-purified anti-Ap^1-17^ Ab was performed as described earlier [22]. Briefly, IVIG were diluted in an equal volume of PBS, repeatedly passed through a BSA-conjugated AffiGel 15 column, and then through an Ap^1-17^-conjugated column; no bound Abs were eluted, indicating the absence of naturally occurring anti-Ap^1-17^ Abs. The nonbound fraction was incubated with protein G-Sepharose and protein A-Sepharose. Phage particles (2×10^11^) were incubated with 30 µl IVIG-coated protein G-Sepharose (first and third rounds) and protein A-Sepharose (second and fourth rounds) to remove isotype- and allotype-specific phage particles. After a 2-h incubation at 4°C, unbound phage particles were recovered and incubated with 30 µl packed protein A-Sepharose (first and third rounds) and protein G-Sepharose (second and fourth rounds) previously coated with 30 µg affinity-purified anti-Ap^1-17^ Abs. After an additional 1-h incubation at 4°C, beads were washed 10 times with Tris-buffered saline (TBS) containing 0.5% Tween 20 to remove unbound phage particles. Bound phage particles were eluted by addition of 500 µl 0.2 M glycine pH 2.8. The eluate was rapidly neutralized with 75 µl 1 M Tris-HCl pH 9.1. Eluted phage particles from each round were amplified in bacteria, purified, and used as input for the next round. After the fourth round, single colonies were selected and amplified.

The supernatants of Ab-selected phage particles were tested for specificity to affinity-purified anti-Ap^1-17^ Abs in an indirect ELISA, using IVIG as a negative control, as previously described [19]. Ab-specific phage clone inserts were sequenced at the M-MEDICAL sequencing facility (Cornaredo, Italy).

Nucleotide sequences were analyzed and motifs were identified as previously described [24]. Other proteins containing the same antigenic motifs were searched for in the UniProtKB/Swiss-Prot Protein Sequence Database using the ScanProsite tool (http://www.expasy.ch/tools/scanprosite/), at the ExPASy Bioinformatics Resource Portal. Proteins expressing the motif were grouped according to taxonomy (*Homo sapiens*, bacteria, fungi and viruses) and apparatus using Bgee: Gene Expression Evolution at the Swiss Institute of Bioinformatics (http://bgee.unil.ch/bgee/bgee).

### Inhibition of phage particle binding by peptide

Microtiter plates were incubated overnight with 5 µg/ml affinity-purified anti-Ap^1-17^ Ab from selected SSc patients. Plates were washed and free protein-binding sites were blocked with PBS-BSA (100 µl/well). Then, 50 µl PBS containing serial dilutions of peptide (starting concentration, 800 µg/ml) was added to the well and incubated for 2 h at 25°C. Then, without removing the inhibitor solution, phage supernatants (50 µl/well; diluted in PBS to the lowest concentration giving 80%–100% of maximal binding to anti-Ap^1-17^ IgG were added and incubated for 2 h. Following three washes with PBS-T20, bound phage particles were detected with HRP-conjugated anti-M13 mAb and o-phenylenediamine, with absorbance read at 490 nm. Results were expressed as the percentage of inhibition of the binding calculated as ([A_490_ in absence of inhibitor-A_490_ in presence of inhibitor]/A_490_ in absence of inhibitor)×100.

### Statistical analyses

Receiver operating characteristics (ROC) analysis was used to find cut-off points which best discriminated ACA^+^ from ACA^−^ groups based on Ap^1-17^ reactivity with patients' sera as described [25]. This analysis was performed using MedCalc software, v. 7.6.0.0, and the discriminating cut-off was automatically obtained. The Mann-Whitney test was used with continuous variables for comparisons between groups. A p value <0.05 indicated statistical significance.

## Results

### Purification and specificity of anti-Ap^1-17^ Abs

**Figure 1:**
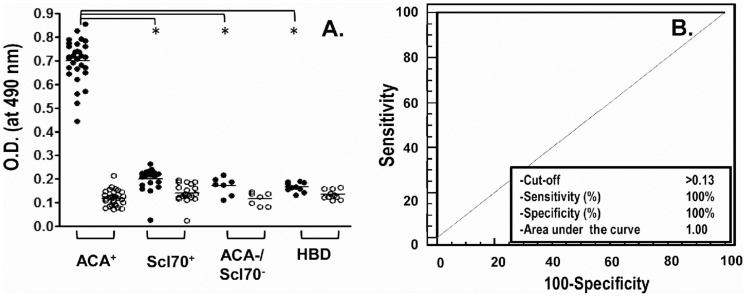
Screening of SSc sera for specificity to Ap^1-17^. (**A.**) Sera from SSc patients (30 ACA^+^, 20 Scl70^+^; 7 ACA^−^/Scl70^−^) were screened for specificity to Ap^1-17^ peptide in an indirect ELISA. Microtiter plates were coated with 5 µg/ml KLH-conjugated Ap^1-17^ (filled circles) or KLH-conjugated Qp1-a (open circles). Wells were incubated for 4 h with serum samples (diluted 1∶100) from the three groups of patients and from 10 healthy blood donors (HBD); samples were tested in duplicate. Bound IgG was revealed with HRP-conjugated anti-human IgG (Fc portion) and *o*-phenylenediamine. Each data point is the mean of duplicate wells (SEM ≤8%). Horizontal lines indicate the mean for each group. O.D., optical density. *Mann-Whitney p<0.0001. (**B**) ROC analysis was performed by including the absorbance binding of each patient's serum to Ap^1-17^ (minus the absorbance of the same sera to the unrelated peptide Qp1-a) as variable, and by comparing the ACA^+^ group to control ACA^−^ group.

Sera from 57 SSc patients with different auto-Ab profiles were screened for binding to Ap^1-17^ in an indirect ELISA (Fig. 1). Only sera from the 30 ACA^+^ patients specifically reacted with Ap^1-17^, giving a mean signal 7-fold higher than that observed with the control peptide (Fig. 1A); this binding signal was also higher than that of serum from the 20 Scl70^+^ patients, the 7 ACA^−^/Scl70^−^ and the 10 HBD (Mann-Whitney, p<0.0001). ROC analysis was used to define the ability of the serum–Ap^1-17^ reactivity to discriminate ACA^+^ patients from the other groups. The absorbance cut-off was 0.13 (Fig. 1B), with 100% sensitivity and 100% specificity, indicating that peptide Ap^1-17^ efficiently discriminates the ACA^+^ group from the ACA^−^ control groups.

**Figure 2:**
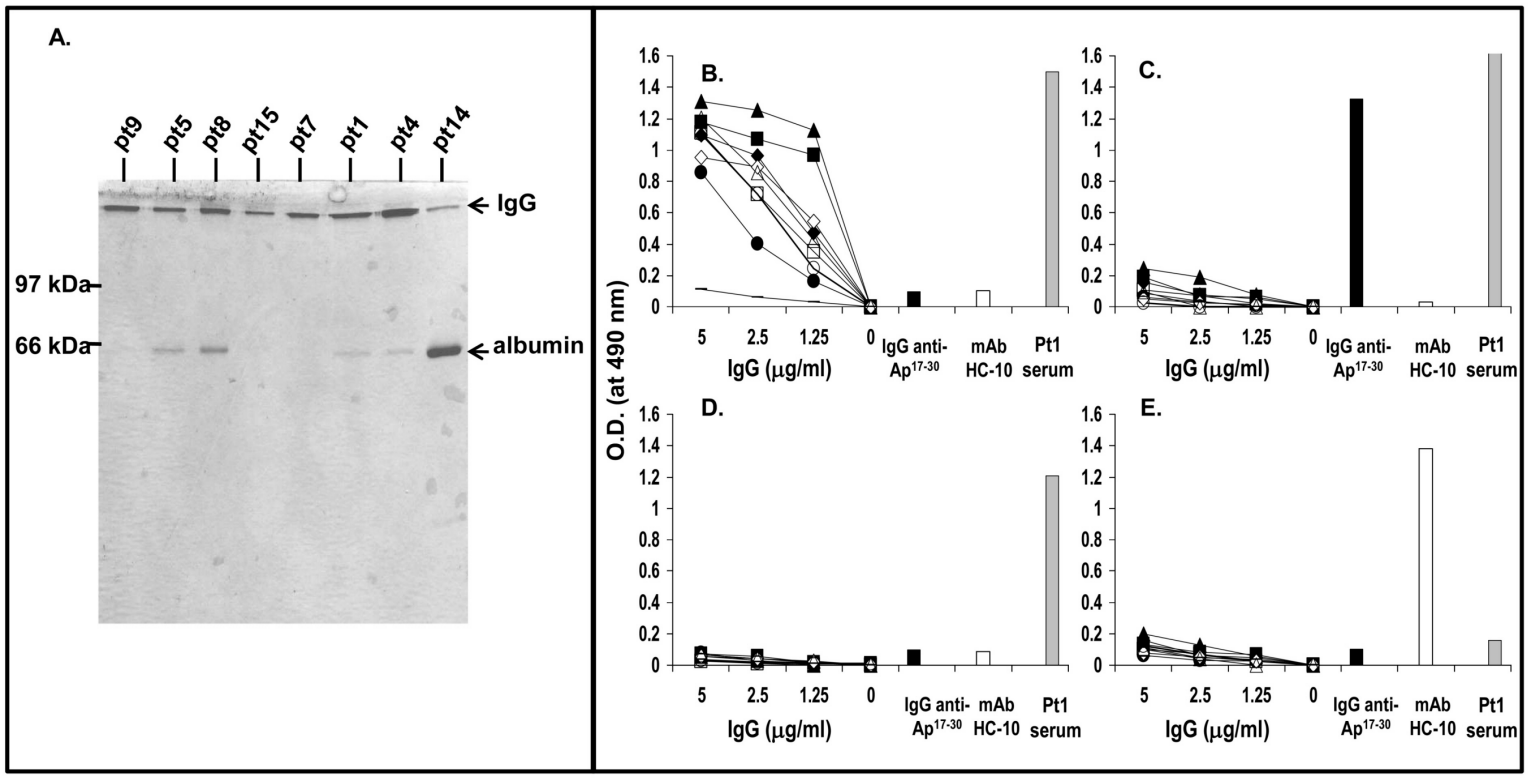
Purity and specificity of anti-Ap^1-17^ Abs purified from SSc patients' serum. (**A.**) Anti-Ap^1-17^ Abs were affinity-purified from serum on Ap^1-17^-conjugated Affi-Gel columns. Purified proteins (2 µg) were separated by SDS-PAGE under non-reducing conditions and stained with Coomassie brilliant blue. (**B–E.**) Indirect ELISAs to determine the binding avidity of affinity-purified anti-Ap^1-17^ Abs from 8 SSc patients. Microtiter plates were coated with 5 µg/ml KLH-conjugated Ap^1-17^ (B.), KLH-conjugated Ap^17-30^ (C.), KLH-conjugated CBp^1-13^ (D.) or KLH-conjugated Qp-1a (E.). Wells were incubated for 4 h with 50 µl PBS containing serial dilutions of anti-Ap^1-17^ Abs from pt1 (♦), pt4 (▪), pt5 (▴), pt7 (•), p8 (◊), pt9 (□), pt14 (▵), or pt15 (○). IVIG (⁃); anti-Ap^17-30^Abs from pt1 (closed bar), and mAb HC-10 (open bar) were used as specificity controls (all at 5 µg/ml). Bound IgG was revealed with HRP-conjugated anti-human IgG or anti-mouse IgG (to detect mAb HC-10 Fc portion) and *o*-phenylenediamine. The coating of the plate with CBp^1-13^ was verified by its reactivity with pt1 serum (gray bar). The data are representative of 2 experiments.

The reactivity with Ap^1-17^ of all sera from ACA^+^ patients indicated that at least a portion of their anti-CENP Abs were directed against the CENP-A protein. Therefore, all of them could be a source of anti-Ap^1-17^ Abs. For convenience, anti-Ap^1-17^ Abs were affinity-purified from the sera of the eight ACA^+^ patients with the highest anti-Ap^1-17^ Ab titers (pt1, pt4, pt5, pt7, pt8, pt9, pt14, and pt15). The eight anti-Ap^1-17^ Abs preparations had variable extents of contamination with human serum albumin (Fig. 2A). The IgG concentration for each preparation was therefore corrected to allow for the percentage of contaminating albumin, calculated following density scanning of Coomassie-stained gels. Following this correction, the Ap^1-17^-specific Ab recovery ranged from 367 µg/ml (pt4) to 84 µg/ml (pt14). The specificity of the preparations for Ap^1-17^ was verified in indirect ELISAs. All anti-Ap^1-17^ Abs dose-dependently reacted with KLH-conjugated Ap^1-17^ (KLH-Ap^1-17^) but displayed differences in relative avidity (expressed as the highest reactivity to peptide at the lowest concentration of IgG) (Fig. 2B). The reactivity was specific since they did not react with KLH-Ap^17-30^ (Fig. 2C), KLH-CBp^1-13^ (Fig. 2D) or KLH-Qp-1a (Fig. 2E). Moreover, IVIG, at the same concentrations, did not react with any peptide while the positive controls anti-Ap^17-30^ Abs and mAb HC-10 gave strong signals with their cognate peptides (KLH-Ap^17-30^, KLH-Qp-1a, respectively). Finally, serum from pt1 bound strongly to the CENP-A-derived peptides Ap^17-30^ and Ap^1-17^, to the CENP-B peptide CBp^1-13^, but not to the Qp-1a peptide.

**Figure 3:**
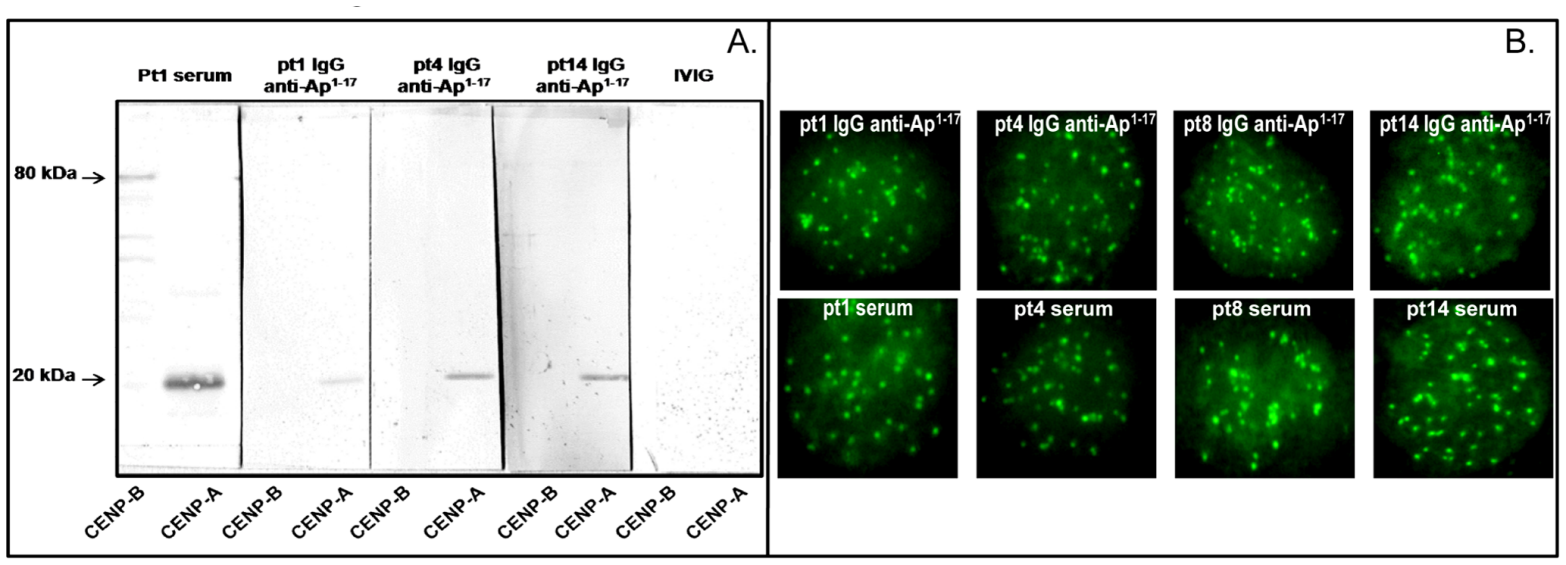
Reactivity of affinity-purified anti-Ap^1-17^ Abs from 3 SSc patients. (**A.**) Recombinant human CENP-B and CENP-A proteins were loaded on alternative lanes (200 ng/lane) of a 12.5% SDS mini-gel under non-reducing conditions, transferred to a PVDF filter, and incubated for 3 h with affinity-purified anti-Ap^1-17^ Abs from pt1, pt4 and pt14. Serum from pt1 and IVIG were used as controls. Bound Abs were detected using HRP-conjugated goat anti-human IgG and diaminobenzidine substrate solution. (**B.**) Centromere staining of pt1, pt4, pt8 and pt14 anti-Ap^1-17^ IgG and of their corresponding serum (1∶100 dilution) to fixed permeabilized HeLa cells. Bound IgG was revealed by fluorescence staining with FITC-conjugated anti-human IgG (Fc portion). Cells were examined with a Nikon confocal microscope and a CCD camera (Nikon digital sight DS-U1), using a 60× Plan Apo VC objective.

The reactivity of affinity-purified anti-Ap^1-17^ Abs to the full length CENP-A protein was then examined by western blotting using recombinant human CENP-A and –B proteins. Six tested samples (pt1, pt4, pt5, pt8, pt9, pt14) gave specific bands at 20 kDa (CENP-A) but no band at 80 kDa (CENP-B); representative results are shown in Fig. 3A. The specificity of the assay was further supported by the binding of serum from pt1 to both CENP-A and CENP-B and by the lack of reactivity of IVIG with both proteins. Similar results were obtained on western blotting in which equimolar amounts of CENP-A and CENP-B were loaded and pt8 serum was used as positive control (Fig. S1). In HeLa cells, the purified anti-Ap^1-17^ Abs retained the ability to bind centromeres and produced a punctuate immunofluorescence pattern over the nucleus (multiple nuclear dots) similar to that observed with whole sera from these SSc patients (Representative results are shown in Fig. 3B).

**Figure 4:**
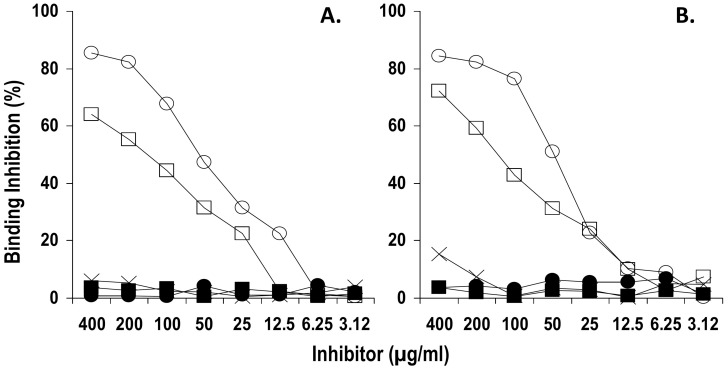
Specificity of anti-Ap^1-17^ IgG for CENP-A documented by human recombinant CENP-A inhibition of anti-Ap^1-17^ IgG binding to KLH-Ap^1-17^. Anti-Ap^1-17^ Abs from pt4 (A) and pt14 (B) were diluted in PBS-T20 at the lowest concentration giving 80%–100% of maximal A_490_ in binding assay, and pre-incubated with an equal volume of PBS containing 2-fold serial dilutions of CENP-A (○), CENP-B (•), and CENP-B-derived peptide CBp^1-13^ (▪). Following a 2-h incubation, the mixture was added to microtiter plate wells coated with KLH-Ap^1-17^. After a 4-h incubation and three washes, bound IgG was detected with HRP-conjugated anti-human IgG (Fc portion) and *o*-phenylenediamine. Inhibition by Ap^1-17^ peptide (□) and by Qp-1a (X) were included as positive and negative controls, respectively. Results are expressed as percentage of binding inhibition. The data are representative of 2 experiments.

The specificity of anti-Ap^1-17^ IgG for CENP-A was further confirmed in an inhibition assay, whereby the binding of anti-Ap^1-17^ IgG to its cognate KLH-peptide was assessed in the presence of different concentrations of inhibitors. Fig. 4 shows that pt4 and pt14 anti-Ap^1-17^ IgG binding was dose-dependently inhibited by CENP-A while CENP-B and the CENP-B peptide CBp^1-13^ did not have any inhibitory effect. The inhibition was specific in that unrelated peptide Qp-1a did not affect the binding, while our positive control Ap^1-17^ did. Overall the data indicate that anti-Ap^1-17^ IgG specifically recognize recombinant CENP-A and the reactivity does not involve the previously defined motif GPxRX expressed on both CENP-A and CENP-B. The concentration of CENP-A giving between 50% and 60% of maximum inhibition (50 µg/ml; 2.5 nmol/ml) was selected for use in inhibition experiments with anti-Ap^1-17^ IgG from pt1, pt5, pt7, pt8, pt9 and pt15. In all cases, binding was inhibited by CENP-A and not by equimolar amounts of CENP-B (Fig. S2).

### Amino acids bound by anti-Ap^1-17^ Abs

The anti-Ap^1-17^ IgG purified from sera of pt4 and pt14 were used to pan the 12-mer PDPL. After panning with pt4 IgG, 40 colonies were randomly selected and ELISA screening showed that 22 clones (55%) reacted specifically with pt4 anti-Ap^1-17^ IgG (giving absorbance values substantially higher than IVIG in assays against immobilized Ap^1-17^). Nucleotide sequencing of 6 of these phage clone inserts identified 6 distinct sequences (Table 1). After panning with pt14 IgG, 82 colonies were randomly selected; ELISA showed that 41 clones (50%) reacted specifically and nucleotide sequencing of all 41 phage clone inserts identified 11 distinct sequences.

**Table 1:** Definition of pt4 and pt14 anti-Ap^1-17^ Abs *motifs*.

Phage clone insert #	Clones, n (%)	Phage clone denomination	Deduced amino acid sequencea)	Binding specificity (A_490 nm_)b)	
				Anti-peptide IgG	IVIGc)
1	1 (4.5)	pc4.10	EMYR**KP**MHAQL**R**----	0.66±0.11	0.045
2	1 (4.5)	pc4.19	--SM**KP**AP**P**VQHQL--	1.72±0.13	0.07±0.06
3	1 (4.5)	pc4.22	--EM**KP**MA**P**IT**R**YT--	1.68±0.1	0.04±0.02
4	1 (4.5)	pc4.26	----**KP**MP**P**MI**R**LVTS	1.58±0.2	0.04±0.01
5	1 (4.5)	pc4.33	GIAK**KP**SA**P**LQ**R**----	1.78±0.16	0.04±0.01
6	1 (4.5)	pc4.40	ASNM**KP**SA**P**MQ**R**----	1.58±0.2	0.04±0.01
pt4 *motif*			----KP--P--R----		
1	18 (43.9)	pc14.3	-IPIP**R**ESI**KP**TW--	1.72±0.1	0.11±0.07
2	1 (2.4)	pc14.4	-DPFT**R**MSI**KP**TG--	1.84±0.12	0.09±0.05
3	4 (9.7)	pc14.6	-DLSP**R**LTI**KP**QR--	1.69±0.08	0.09
4	1 (2.4)	pc14.8	-DPMS**R**ITM**KP**HI--	2.01±0.11	0.12±0.05
5	1 (2.4)	pc14.9	-LPWI**R**TTE**KP**QF--	1.9	0.11±0.05
6	1 (2.4)	pc14.49	DHPQT**R**TAP**KP**V---	2.1±0.12	0.11±0.02
7	1 (2.4)	pc14.56	-DPHY**R**NSP**KP**DS--	1.75±0.1	0.14
8	1 (2.4)	pc14.61	---NY**R**ETP**KP**TWPT	1.86±0.12	0.11
9	2 (4.8)	pc14.72	---EF**R**SSV**KP**QHPL	1.7±0.2	0.14±0.3
10	10 (24.3)	pc14.74	-AHDH**R**SSL**KP**TR--	1.82±0.1	0.15±0.02
11	1 (2.4)	pc14.75	-QGHM**R**QTA**KP**FV--	1.8±0.1	0.15±0.01
pt14 *motif*			-----R-s-KP---- t		

a) Multiple alignments were performed with MULTALIN at Pole Bio-Informatique Lyonnaise (http://npsa-pbil.ibcp.fr/cgi-bin/npsa_automat.pl?page=/NPSA/npsa_multalin.html). Amino acids matching those of the motif are underlined and bold.

b) Phage supernatants were diluted 16-fold. Values are mean (±SEM) of duplicate wells. Results are representative of two experiments.

c) IVIG, intravenous human immunoglobulins for human use.

Shown are phage clone inserts identified by panning a 12-mer linear phage display peptide library with affinity-purified anti-Ap^1-17^ Abs from pt4 and pt14 patients with SSc, who expressed Abs to centromere-associated protein (CENP-A) and to its peptide Ap^1-17^ (MGPRRRSRKPEAPRRRS). Reported for each phage clone insert are the deduced amino acid sequence and the binding specificity of the phage supernatant to Ap^1-17^, as determined in comparison to IVIG in indirect ELISAs.

**Figure 5:**
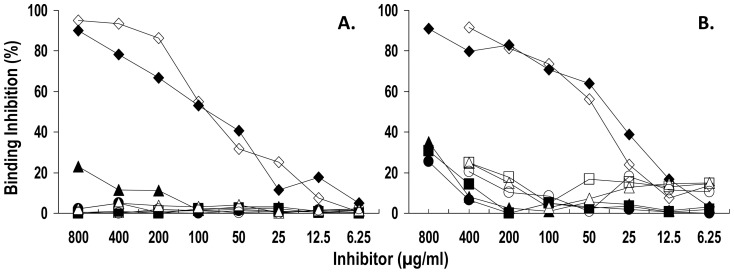
Specific peptide inhibition of phage particle binding to anti-Ap^1-17^ IgG demonstrates that phage insert sequences recognize the antigen-combining sites of anti-Ap^1-17^ IgG. Microtiter plates were coated with affinity-purified anti-Ap^1-17^ IgG from pt4 (A.) or pt14 (B.). After the blocking of free protein-binding sites with PBS-BSA, wells were incubated for 2 h with 50 µl PBS containing serial dilutions of either free (closed symbols) or KLH-conjugated (open symbols) Ap^1-17^ (rhombus), Ap^17-30^ (triangle), CBp^1-13^ (circle) or Qp-1a (square). Then, without removing the inhibitor, 50 µl/well of an appropriate dilution of phage supernatant pc4.33 (A.) and pc14.49 (B.) was added and incubation prolonged for 2 h. Bound phage particles were detected by sequential addition of HRP-anti-M13 mAb and o-phenylenediamine solution. Results are expressed as percentage of binding inhibition. Each data point is the mean of duplicate wells. The data are representative of two experiments. The maximum amount of inhibitor (800 µg) corresponds to 382 nmol Ap^1-17^, 558 nmol Ap^17-30^, 486 nmol CBp^1-13^ and 574 nmol Qp-1a.

To ensure that the selected phage clone insert sequences recognized the antigen-combining site of anti-Ap^1-17^ IgG, the binding of phage particles to anti-Ap^1-17^ IgG was assessed in the presence of free and KLH-conjugated peptide. Ap^1-17^ and KLH-Ap^1-17^ dose-dependently inhibited the binding of pc4.33 (Fig. 5A) and pc14.49 (Fig. 5B) supernatants to pt4 and pt14 anti-Ap^1-17^ IgG, respectively. The inhibition was specific since Ap^17-30^, CBp^1-13^ and Qp-1a did not have any inhibitory effect. The results indicate that phage insert sequence is complementary to the Ag-combining site of anti-Ap^1-17^ IgG and that peptide conformation is not influenced regardless of whether peptide is presented on phage PIII protein or coupled to KLH.

Alignment of the 6 sequences from pt4 identified the motif KPxxPxxR (Table 1), which in turn aligned with the amino acids K^9^ to R^16^ of Ap^1-17^ (Table 2). The ^9^KP^10^ dipeptide was expressed by all the sequences; it associated with P^13^ and R^16^ in 5 sequences (83.3%) and with A^12^ in 3 sequences (50%). These results show that ^9^KP^10^, P^13^ and R^16^ are the most critical amino acids for the interaction with IgG anti-Ap^1-17^ from pt4. Regarding pt14, alignment of the 11 insert sequences identified the motif Rxs(t)xKP, which matched R^5^ to P^10^ of Ap^1-17^. Expression of R^5^ and ^9^KP^10^ by all the sequences indicated that they are the most critical amino acids for the interaction with this patient's anti-Ap^1-17^ IgG, whereas S^7^ could be used interchangeably with the chemically similar T^7^. Finally, alignment of both patients' motifs with Ap^1-17^ showed that ^9^KP^10^ are the most critical amino acids for the interaction with anti-Ap^1-17^ IgG from SSc patients (**Table 2**).

**Table 2:** Alignment of pt4 and pt14 deduced motifs with CENP-A protein sequence.

Denomination	Amino acid sequence
pt4 motif	--------**KP**--**P**--**R**-
pt14 motif	----**R**-s-**KP**----
CENP-A^1-17^	MGPR**R**R**S**R**KP**EA**P**RR**R**S
Consensus	----**R**-s-**KP**--**P**—-**R**-

Motif amino acid “S” shown in small letter could be used interchangeably with “T”.

### Reactivity profiles of SSc patients' anti-Ap^1-17^ Abs with anti-CENP-A motifs

**Figure 6:**
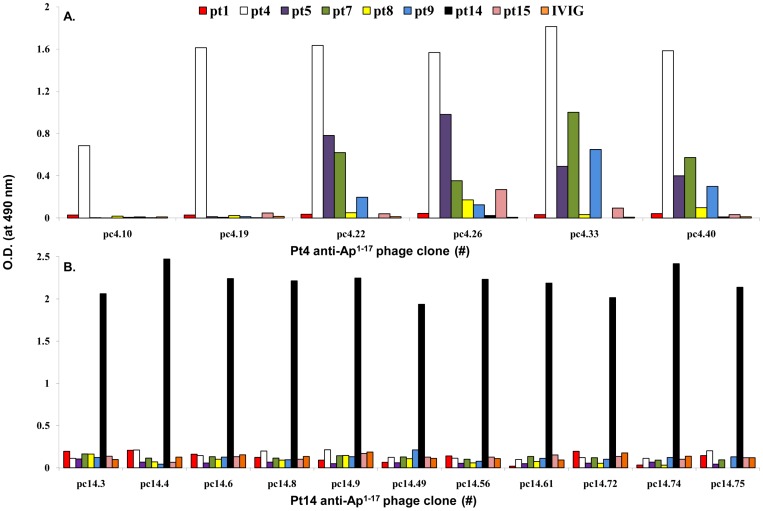
Anti-Ap^1-17^ Abs from different SSc patients recognize different motifs within the same peptide. Microtiter plates were coated with 10 µg/ml anti-Ap^1-17^ Abs from 8 SSc patients or with IVIG as negative control. Supernatants from pt4 (Panel A) and pt14 (Panel B) phage clones were diluted 16-fold in PBS and added to the wells (100 µl/well). After a 4-h incubation, the Ab-phage clone interaction was detected with HRP-conjugated anti-M13 mAb and *o*-phenylenediamine. Each data point is the mean of duplicate wells. The data are representative of two experiments.

To examine the cross-reactivity between the peptide sequences identified by PDPL panning using anti-Ap^1-17^ IgG from two SSc patients and the IgG of the other 6 SSc patients, a series of indirect ELISAs was performed (Fig. 6). Assessment of the reactivity of the 6 pt4 phage clones (pc4.X) and the 11 pt14 phage clones (pc14.X) with the patients' anti-Ap^1-17^ IgG allowed us to distinguish the patients into groups on the basis of their immunoreactivity profiles. First, IgG from pt4 reacted with all pc4.X (Fig. 6A) but with no pc14.X. Conversely, IgG from pt14 reacted with all pc14.X (Fig. 6B) but with no pc4.X. A third group included pt5, pt7 and pt9 IgG reacting, though to a variable extent, with pc4.22, pc4.26, pc4.33 and pc4.40 (Fig. 6A). Finally, a fourth group included pt1, pt8 and pt15 IgG which gave low or no signal in all ELISAs (Fig. 6A, 6B). The specificity of the assay was confirmed by the lack of reactivity of IVIG. These results indicate that anti-Ap^1-17^ IgG from different patients display unique specificities despite their recognition of the same peptide.

### Presence of anti-Ap^1-17^ Ab antigenic motifs in other proteins

The Swiss-Prot database was scanned to determine if the two antigenic motifs were present in other human or pathogenic proteins. This search highlighted the large number of different types of proteins expressing the motifs recognized by pt4 and pt14 IgG. In *Homo sapiens*, a total of 941 proteins matched the pt4 motif (KPxxPxxR) while 817 had the pt14 motif (Rxs(t)xKP). Similar large numbers were observed in bacteria (785 and 853, respectively). However, lower numbers of matching proteins were found in fungi (137 and 176) and in viruses (66 and 32).

To examine the types of proteins possibly targeted by anti-Ap^1-17^ IgG, the matching human proteins were sorted according to the organ systems (apparatuses) in which they are expressed (Table 3). High numbers (>90) of pt4 and pt14 motif-containing proteins were detected in the central nervous system, cardiovascular system and gastrointestinal system, while low numbers (<40) were detected in the peripheral nervous system, in the cutaneous and hematopoietic apparatuses, and in the mesothelium. Overall, there was a strong correlation between the numbers of proteins with the two motifs in the different apparatuses (linear regression R^2^ = 0.7). Regarding specific tissues, it is interesting to note that for the pineal gland, optical nerve, liver, cartilage, ovary, inner ear, iris and lens no or very few (≤2) proteins were found to either motifs. For the other tissues, the ratio of numbers of pt4 to pt14 motif-containing proteins was highest in muscle (ratio = 3), blood (2.33), bone (2.14), and lung (1.78) and lowest in ciliary body (0.13), dorsal root ganglion (0.25), hypophysis (0.27), myocardium (0.4) and diencephalon (0.44). Finally, qualitative analysis of the proteins showed that pt4 and pt14 motifs retrieved two different sets of proteins for each specific tissue (data not shown). This molecular analysis demonstrates that there is a wide motif-sequence distribution among species and human tissues, and it suggests that the motifs are ancestral conserved. These results suggest, therefore, that anti-CENP-A^1-17^ IgG may cross-react with a wide variety of proteins in different human tissues and from different pathogenic species.

**Table 3:** Numbers of human proteins expressing the pt4 and pt14 antigenic motifs, by organ system (apparatus) and specific tissue.

Apparatus	Specific tissue	No. of proteins expressing the motifa)	
		pt4 - KPXXPXXR	pt14 – RXS(T)XKP
**Central nervous**	**All**	**122**	**172**
	*Brain; Diencephalon; Pineal gland; Cerebellum; Medulla oblongata*	*52; 32; 0; 35; 3*	*36; 72; 0; 55; 9*
**Peripheral nervous**	**All**	**15**	**41**
	*Dorsal root ganglion; Optical nerve; Spinal cord*	*9; 0; 5*	*35; 1; 5*
**Cardiovascular**	**All**	**137**	**134**
	*Heart; Endocardium; Myocardium; Artery; Vein*	*34; 27; 12; 41; 23*	*36; 24; 30; 27; 17*
**Endocrine**	**All**	**53**	**46**
	*Hypophysis; Thyroid; Adrenal Gland; Pancreas*	*3; 15; 14; 21*	*11; 11; 11; 13*
**Cutaneous**	**All**	**22**	**28**
	*Dermis; Epidermis*	*0; 0*	*0; 20*
**Gastrointestinal**	**All**	**168**	**112**
	*Oral cavity; Esophagus; Gut; Liver; Gall bladder*	*61; 19; 86; 2; 0*	*38; 15; 52; 2; 5*
**Hematopoietic**	**All**	**34**	**17**
	*Thymus; Blood; Spleen*	*14; 7; 13*	*9; 3; 5*
**Osteoarticular**	**All**	**69**	**33**
	*Bone; Cartilage; Joint; Muscle*	*30; 0; 18; 21*	*14; 0 ;12; 7*
**Reproductive**	**All**	**99**	**69**
	*Ovary; Testis*	*0; 9*	*1; 11*
**Respiratory**	**All**	**62**	**35**
	*lung*	*50*	*28*
**Sensory**	**All**	**78**	**76**
	Inner ear	*0*	*0*
	Eye *(Ciliary body; Iris/lens; Retina)*	*24 (3; 0; 16)*	*15 (22; 0; 13)*
	Olfactory organ	35	26
**Urogenital**	**All**	**46**	**26**
**Mesothelium**	**All**	**36**	**28**

a) The number of proteins expressing the pt4 and pt14 motifs were determined by searching in the Swiss-Prot database (http://prosite.expasy.org/scanprosite/), using human tissue expression (Bgee data) filters.

## Discussion

This study used affinity-purified anti-Ap^1-17^ Abs from two patients with SSc in PDPL panning experiments to define the fine specificity of Abs to the NH_2_-terminal immunodominant epitope of CENP-A. Three lines of evidence indicate that peptide Ap^1-17^ effectively mimics recombinant CENP-A and is efficient in purifying antibodies relevant to the proposed study. First, Ap^1-17^ reacted almost exclusively with sera from patients in the ACA^+^ group (all identified on a routine basis with anti-CENP-B ELISA), efficiently discriminating this group from the ACA^−^ control groups, and reproducing the high concordance rate (94.3%) of sera reactivity to either CENP-A (Ap^1-17^ in our study) or CENP-B previously reported in a larger cohort of ACA^+^ SSc patients [26]. Second, the IgG purified with Ap^1-17^ specifically reacted with the cognate peptide and human recombinant CENP-A and not with Ap^17-30^, recombinant CENP-B and CBp^1-13^; it also produced a centromeric punctuate pattern in immunofluorescence assay similar to that of whole sera. The nuclear dots obtained with some anti-Ap^1-17^ IgG were not as sharp as those obtained with serum, probably due to the recognition of only one specific CENP-A epitope rather than multiple epitopes. Lastly, the coupling of KLH to Ap^1-17^ did not change its antigenic properties in terms of conformation or structure, since free peptide and KLH-conjugated peptide both competed for the binding of anti-Ap^1-17^ IgG to KLH-Ap^1-17^.

Analysis of Ab-selected phage clones expressing 12-mer linear peptides allowed us to identify two specific, partially overlapping motifs: the pt4 motif ^9^KPxxPxxR^16^ and the pt14 motif ^5^Rx(ST)xKP^10^. Amino acids K^9^ and P^10^ are the most critical for binding in that they are present at the same position of all peptides selected with pt4 and pt14 IgG. Despite the overlapping nature of the motifs, there was no cross-reactivity between one patient's IgG and the other's motif. Furthermore, there was limited cross-reactivity with the other SSc patients' IgG: some pt4-specific phage clones (pc4.22, pc4.26, pc4.33 and pc4.40) were recognized to a variable extent by anti-Ap^1-17^ IgG from pt5, pt7 and pt9, while none of the 11 pt14-specific clones reacted with the other patients' anti-Ap^1-17^ IgG. These results indicate that although pt4 and pt14 IgG recognize the same CENP-A segment, they have unique specificities.

One might argue that these unique specificities only reflect the recognition by phage particle inserts (phage PIII protein-fused peptide) of antigenic determinants outside the antigen-combining site of anti-Ap^1-17^ IgG, as demonstrated in another Ag/Ab system using anti-idiotypic mAb [27]. This possibility was excluded by the ability of Ap^1-17^ to specifically inhibit the binding of phage clone particles to anti-Ap^1-17^ IgG. As a similar inhibitory effect was obtained using the same peptide conjugated to KLH, it is reasonable to conclude that peptide conformation was not influenced regardless of whether it was free or coupled to KLH, both forms of peptide competing with the binding of phage protein III-coupled peptides. Furthermore, the specificity of phage clone particles was not influenced by the possible presence of naturally occurring auto-anti-Ap^1-17^ Abs [28], [29] in IVIG, used during PDPL panning to remove phage particles specific for isotypic and allotypic determinants. Indeed, IVIG did not react in ELISA with Ap^1-17^ and no anti-Ap^1-17^ Abs could be affinity purified from IVIG. The absence of naturally occurring anti-Ap^1-17^ Abs in IVIG suggests that these Abs are more likely induced by a T cell-mediated, Ag-driven immune response, in agreement with the association between the presence of ACA in SSc patients and polar amino acids at position 26 of HLA-class II allele DQB1 [30], [31].

The fact that no pt4 or pt14 phage clones reacted with pt1, pt8 and pt15 anti-Ap^1-17^ IgG suggests that additional motifs may be involved in the binding of these IgG with CENP-A. It is unlikely that these three patients' anti-Ap^1-17^ IgG recognize the motif G/APXRX, previously identified by means of mutational analysis [16], [18] and found to be expressed both by CENP-B (^2^GPKRR^6^) [18] and by CENP-A in its two immunodominant epitopes (Ap^1-17^ and Ap^17-30^). Indeed, pt1 and pt8 IgG did not react with CENP-B in western blotting nor did pt1, pt8 and pt15 IgG bind human recombinant CENP-B, CBp^1-13^ and Ap^17-30^ (also expressing the motif G/APXRX) in ELISA (binding and inhibition). These data confirm our previous findings in which anti-CENP Abs specific for the Ap^17-30^ portion of CENP-A displayed no cross-reactivity with CENP-B [19]. Altogether, these results strongly suggest that CENP-A Abs are generated independently from anti-CENP-B Abs and that the previously reported motif G/APXRX is not responsible for any cross-reactivity between anti-CENP-A and -CENP-B Abs [18], [32]. Supporting this conclusion are the results by Mahler *et al.* [33], who reported a lack of cross-inhibition between anti-CENP-A and anti-CENP-B Abs, using an amino terminal CENP-A-derived peptide (sequence not reported) and recombinant CENP-B as inhibitor.

The identification of these two novel and partially overlapping motifs, the differential cross-reactivity of pt5, pt7 and pt9 IgG with some pt4-specific phage clones, and the lack of reactivity of seven patients' IgG preparations with any pt14-specific phage clones together suggest that there is great heterogeneity in the fine specificity of anti-Ap^1-17^ IgG from patient to patient. Given the great diversity in their specificity, it is possible that these IgG are highly somatically mutated, probably as the result of a constant antigenic selective pressure. Furthermore, it will be interesting to search for any correlation between the fine specificity of these Abs and any single disease manifestation. Indeed, Tamby et al. [34] reported an association between the presence of Abs directed to an unknown 40 kDa protein and pulmonary arterial hypertension in patients with idiopathic and scleroderma-associated pulmonary hypertension. Accordingly, since the present investigation identified 4 different profiles based on the IgG reactivity (or lack of reactivity) with the pt4 and pt14 motifs, it will be interesting to assess the possible associations of each reactivity profile with the extent of cutaneous and gastrointestinal involvement and with the presence (or absence) of pulmonary hypertension (or fibrosis). If successful, this approach should provide more specific predictive and prognostic clinical information than is currently possible through the detection of anti-CENP-A and –CENP-B Abs using the whole recombinant proteins in ELISA or LIA, which appeared only to slightly increase the diagnostic sensitivity for SSc compared to the detection of anti-CENP-B only [12], [26].

Finally, this study permits the development of a model to explain the phenomenon of epitope spreading [35]. This phenomenon occurs when Abs, either naturally occurring in the sera [32] or raised following peptide immunization [36], cross-react with another region of the same protein (intra-molecular epitope spreading) [32], [37] or even with a different protein (inter-molecular epitope spreading) [32], [36]. The pt4 and pt14 IgG analyzed here both recognize the same CENP-A-derived peptide (Ap^1-17^). Even so, because they recognize different motifs, they may cross-react with two completely different sets of proteins. This hypothesis is supported by the fact that scanning the UniProtKB database using the two motifs as search terms retrieved two completely different sets of proteins with wide interspecies and human tissue distributions. Future investigations will hopefully define the proteins that effectively are involved in priming anti-CENP-A Ab.

## Supporting Information

Figure S1 **Western blot analysis of the reactivity of pt8, pt4 and pt14 to equimolar amounts of human recombinant CENP-A and CENP-B, to document anti-Ap^1-17^ IgG recognition of recombinant human CENP-A but not CENP-B.** Equimolar amounts (25 pmol) of CENP-A (500 ng/lane) and CENP-B (2 µg/lane) (Abcam, Cambridge, UK) were separated by 12.5% SDS-PAGE under non-reducing conditions and transferred onto a polyvinylidene fluoride membrane (PVDF) previously soaked in absolute methanol. After blockade of free protein-binding sites by a 2-h incubation in PBS-BSA, the filter was incubated with pt8, pt4 and pt14 anti-Ap^1-17^ IgG (2 µg/ml) for 3 h at 25°C with gentle shaking. Bound Ig was detected by the sequential addition of HRP-conjugated xeno-Abs to human IgG (Fc portion) and diaminobenzidine. Pt8 serum was used as positive control. The efficiency of proteins transferred was assessed by Coomassie Brilliant blue staining of CENP-A and CENP-B on a parallel track of PVDF.
(TIF)

Figure S2 **Specificity of anti-Ap^1-17^ IgG for CENP-A documented by human recombinant CENP-A inhibition of pts anti-Ap^1-17^ IgG binding to KLH-Ap^1-17^.** Anti-Ap^1-17^ Ab preparations from eight patients were diluted in PBS-T20 at the lowest concentration giving 80%–100% of maximal A_490_ in the binding assay, and pre-incubated with an equal volume of PBS containing 2.5 nmol/ml human recombinant CENP-A (closed bars) and CENP-B (open bars). Following a 2-h incubation, the mixture was added to microtiter plate wells coated with KLH-Ap^1-17^. After a 4-h incubation and three washes, bound IgG was detected with HRP-conjugated anti-human IgG (Fc portion) and *o*-phenylenediamine. Results are expressed as the percentage of binding inhibition. The data are representative of 2 experiments.
(TIF)
