# Supplementary figures and images for: Autoantibodies Recognizing the Amino Terminal 1-17 Segment of CENP-A Display Unique Specificities in Systemic Sclerosis
Source: PLoS One. 2013 Apr 22;8(4):e61453. doi: 10.1371/journal.pone.0061453 (PMC3632547; doi:10.1371/journal.pone.0061453)

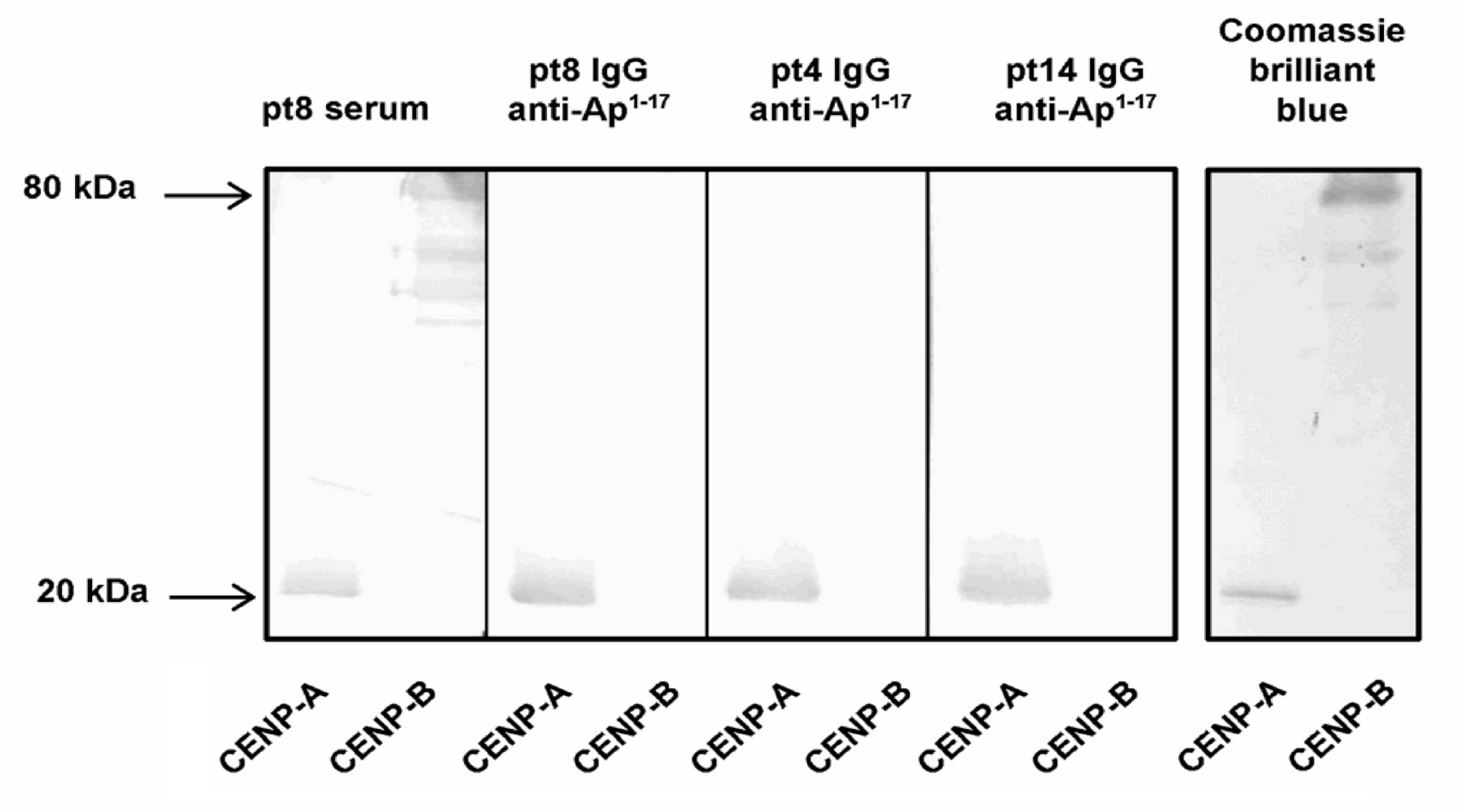

Supplement: Figure S1 — Western blot analysis of the reactivity of pt8, pt4 and pt14 to equimolar amounts of human recombinant CENP-A and CENP-B, to document anti-Ap1-17 IgG recognition of recombinant human CENP-A but not CENP-B. Equimolar amounts (25 pmol) of CENP-A (500 ng/lane) and CENP-B (2 µg/lane) (Abcam, Cambridge, UK) were separated by 12.5% SDS-PAGE under non-reducing conditions and transferred onto a polyvinylidene fluoride membrane (PVDF) previously soaked in absolute methanol. After blockade of free protein-binding sites by a 2-h incubation in PBS-BSA, the filter was incubated with pt8, pt4 and pt14 anti-Ap1-17 IgG (2 µg/ml) for 3 h at 25°C with gentle shaking. Bound Ig was detected by the sequential addition of HRP-conjugated xeno-Abs to human IgG (Fc portion) and diaminobenzidine. Pt8 serum was used as positive control. The efficiency of proteins transferred was assessed by Coomassie Brilliant blue staining of CENP-A and CENP-B on a parallel track of PVDF. (TIF) [file pone.0061453.s001.tif]

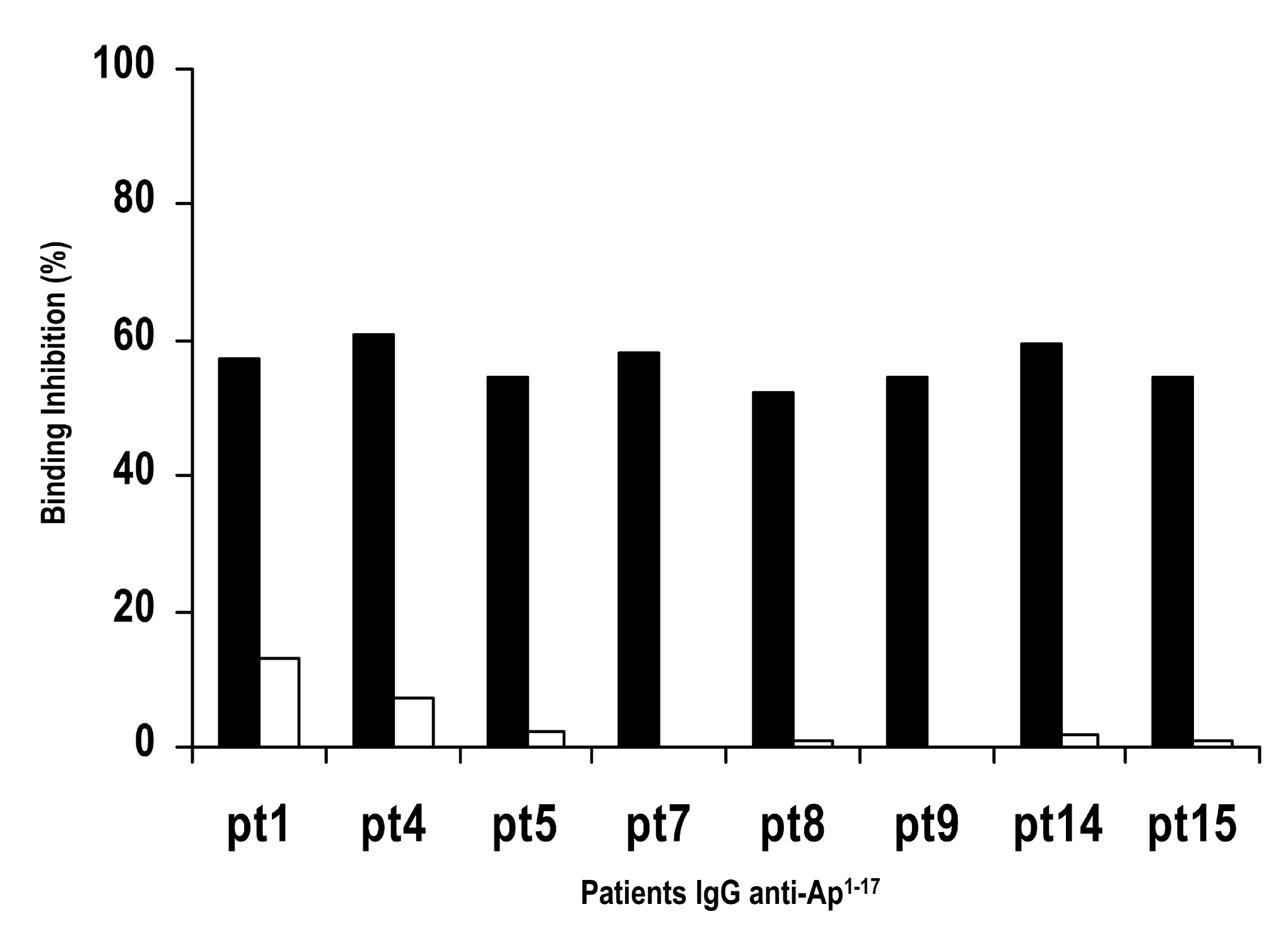

Supplement: Figure S2 — Specificity of anti-Ap1-17 IgG for CENP-A documented by human recombinant CENP-A inhibition of pts anti-Ap1-17 IgG binding to KLH-Ap1-17. Anti-Ap1-17 Ab preparations from eight patients were diluted in PBS-T20 at the lowest concentration giving 80%–100% of maximal A490 in the binding assay, and pre-incubated with an equal volume of PBS containing 2.5 nmol/ml human recombinant CENP-A (closed bars) and CENP-B (open bars). Following a 2-h incubation, the mixture was added to microtiter plate wells coated with KLH-Ap1-17. After a 4-h incubation and three washes, bound IgG was detected with HRP-conjugated anti-human IgG (Fc portion) and o-phenylenediamine. Results are expressed as the percentage of binding inhibition. The data are representative of 2 experiments. (TIF) [file pone.0061453.s002.tif]
